# Developing a Health Information Technology Systems Matrix: A Qualitative Participatory Approach

**DOI:** 10.2196/jmir.6499

**Published:** 2016-10-06

**Authors:** Jolie N Haun, Margeaux Chavez, Kim M Nazi, Nicole Antinori

**Affiliations:** ^1^ HSR&D Center of Innovation on Disability and Rehabilitation Research James A. Haley VA Hospital US Department of Veterans Affairs Tampa, FL United States; ^2^ Department of Community & Family Health College of Public Health University of South Florida Tampa, FL United States; ^3^ Veterans and Consumers Health Informatics Office Veterans Health Administration Department of Veterans Affairs Albany, NY United States

**Keywords:** veterans, patient-centered care, information resources, patient preferences, integrated delivery system, health information technology

## Abstract

**Background:**

The US Department of Veterans Affairs (VA) has developed various health information technology (HIT) resources to provide accessible veteran-centered health care. Currently, the VA is undergoing a major reorganization of VA HIT to develop a fully integrated system to meet consumer needs. Although extensive system documentation exists for various VA HIT systems, a more centralized and integrated perspective with clear documentation is needed in order to support effective analysis, strategy, planning, and use. Such a tool would enable a novel view of what is currently available and support identifying and effectively capturing the consumer’s vision for the future.

**Objective:**

The objective of this study was to develop the VA HIT Systems Matrix, a novel tool designed to describe the existing VA HIT system and identify consumers’ vision for the future of an integrated VA HIT system.

**Methods:**

This study utilized an expert panel and veteran informant focus groups with self-administered surveys. The study employed participatory research methods to define the current system and understand how stakeholders and veterans envision the future of VA HIT and interface design (eg, look, feel, and function). Directed content analysis was used to analyze focus group data.

**Results:**

The HIT Systems Matrix was developed with input from 47 veterans, an informal caregiver, and an expert panel to provide a descriptive inventory of existing and emerging VA HIT in four worksheets: (1) access and function, (2) benefits and barriers, (3) system preferences, and (4) tasks. Within each worksheet is a two-axis inventory. The VA’s existing and emerging HIT platforms (eg, My HealtheVet, Mobile Health, VetLink Kiosks, Telehealth), My HealtheVet features (eg, Blue Button, secure messaging, appointment reminders, prescription refill, vet library, spotlight, vitals tracker), and non-VA platforms (eg, phone/mobile phone, texting, non-VA mobile apps, non-VA mobile electronic devices, non-VA websites) are organized by row. Columns are titled with thematic and functional domains (eg, access, function, benefits, barriers, authentication, delegation, user tasks). Cells for each sheet include descriptions and details that reflect factors relevant to domains and the topic of each worksheet.

**Conclusions:**

This study provides documentation of the current VA HIT system and efforts for consumers’ vision of an integrated system redesign. The HIT Systems Matrix provides a consumer preference blueprint to inform the current VA HIT system and the vision for future development to integrate electronic resources within VA and beyond with non-VA resources. The data presented in the HIT Systems Matrix are relevant for VA administrators and developers as well as other large health care organizations seeking to document and organize their consumer-facing HIT resources.

## Introduction

The strategic plan of the US Department of Veterans Affairs (VA) aims to provide a modern, consistent health service experience to put veterans in control of how, when, and where they wish to be served [1,2]. The VA’s health information technology (HIT) apps and systems, such as VetLink Kiosks, the My HealtheVet patient and provider portal, and Web and mobile solutions, are central to the realization of the VA strategic plan because these services support an integrated patient experience across multiple technology platforms [3]. Comprehensive and integrated HIT that is based on patient preferences in various contexts is shown to have meaningful effects on patient engagement, empowerment, quality of care, and health outcomes [4,5]. Although the VA’s investment in HIT supports patient self-care management, improved patient-provider communication, and better patient health outcomes, the adoption and sustained use of these technologies varies widely among veteran patients and VA providers. Reasons for this variance in use include lack of awareness of available resources; lack of skills, experience, and/or motivation to use technology effectively; and discrepancies in how these technologies meet the specific needs of targeted users [6,7].

An aim of this VA-funded research was to develop a novel multiaxis Health Information Technology (HIT) Systems Matrix including currently available and desired future VA patient-facing platforms, their features, availability, and conditions for appropriate use. Although extensive system documentation exists for various VA HIT systems, a more centralized and integrated perspective with clear documentation is needed in order to support effective analysis, strategy, planning, and use. Such a tool would enable a novel view of what is currently available and support identifying and effectively capturing consumers’ vision for the future. The HIT Systems Matrix was developed with input from participants and expert panel members to represent relevant topics, such as access, function, preferences, barriers to use, and relevant user tasks. Topic-related data were organized using a matrix because, although it is treated as a single entity, diverse content can be documented and presented in a systematic way using rows and columns for ease of readability and matrix use.

In this paper, we describe our protocol and product development process that leveraged a participatory approach to cultivating an inventory of the current HIT system used by the VA. Stakeholder groups and veteran informants were encouraged to include their vision for the future of VA’s tethered system of electronic resources.

## Methods

The HIT Systems Matrix is the first inclusive inventory of VA’s electronic health resources. It was developed in partnership with a panel of subject matter experts, operational and clinical stakeholders, and veteran/caregiver focus group participants. To develop the HIT Systems Matrix, expert panel members and veteran focus group participants provided initial descriptive information about VA and non-VA electronic health resources veterans may utilize for health care management. The protocol for this study has been previously published and can be referenced for a detailed description of the methods [8].

### Sample and Sampling

#### Veteran Sample

In qualitative research such as this, sample size relies on the quality and richness of information obtained [9,10]. We purposively recruited 47 veterans and one caregiver as “informants” who were English speaking, aged 35 years and older, had at least two chronic comorbid conditions (eg, diabetes and high blood pressure), and were invested in using HIT, measured by use of two or more electronic resources or VA HIT more than once a month. Exclusion criteria included veterans with visual, hearing, or cognitive impairments that would prevent consent and full study participation. Participants were recruited for study participation until domain and theme saturation was reached. Veterans received up to US $50 for their participation (US $25 for participating in each focus group). This study was approved and regulated by the VA Central Institutional Review Board.

#### Expert Panel

Snowball sampling was used to identify providers, key operational representatives, and subject matter experts who could serve as expert panel members. Initial invitations were sent via email to operational partners who were asked to assess gaps in representation and nominate other experts or stakeholders to participate. Over the course of 6 months, expert panel members were asked to participate in monthly HIT Systems Matrix development meetings, which culminated in the pairwise comparison activity [11]. As indicated by VA regulations, panel members participated as employees during their regular work schedule and were not compensated by the study.

### Data Collection

Researchers used teleconference technology to collect initial descriptive data about VA electronic resources from expert panel members (n=34) to inform the development of the HIT Systems Matrix. Electronic mail was used to obtain individual panel member’s responses to the structured pairwise comparison activity. A total of 13 expert panel members completed the pairwise comparison activity. A total of 48 participants provided initial descriptive data about VA and non-VA HIT during the first round of focus groups and a total of 21 completed the pairwise comparison activity.

Expert panel members and veterans were asked to provide information about each VA HIT system, including accessibility, function, and the perceived benefits and barriers related to using VA HIT. Participants were asked to supply additional descriptive information regarding their preferences for using VA HIT in general and complete specific health care management tasks. Additionally, veterans were also asked to provide information about the accessibility, function, and use of commonly used non-VA electronic health resources. Focus groups were conducted with veteran participants to complete the pairwise comparison process.

### Data Management and Analysis

The VA HIT Systems Matrix was developed iteratively. The first step was identifying the patient-facing HIT available to veterans, their features, and elements for prioritization. The research study team identified relevant VA HIT platforms (eg, My HealtheVet, Mobile Health, VetLink Kiosks, Telehealth), features (eg, secure messaging, Blue Button, prescription refill), and elements for prioritization (eg, access/availability, specific resources, user groups, and context). This initial activity facilitated a focus on available HIT and their functions and features; the tool was revised throughout this process, particularly as data were collected from expert panel members and veterans in subsequent steps of the process. Table 1 provides a draft sample of the VA HIT Systems Matrix on completion of the first step of the development process.

The second step focused on expanding content developed in the first step through an information gathering process with expert panel members. To complete this second step, we developed the initial model representing a detailed inventory of platforms, their features, characteristics, and contexts for use. The multiaxis HIT Systems Matrix included both existing and future (planned or desired) VA patient-facing platforms, their features, availability, and conditions for appropriate use. Due to the complexity of VA HIT and the elements of interest, the HIT Systems Matrix was developed using an Excel workbook with several worksheets representing relevant topics, such as access, function, preferences, barriers to use, and relevant user tasks.

In the third step, we integrated data collected during veteran/caregiver focus groups to represent this user perspective. Focus group data was analyzed using content analysis. Directed content analysis allowed the team to focus on the core elements addressed in the focus group script items to identify patterns in descriptions of experience, behavior, and beliefs so that the phenomena could be understood within context [12]. Focus group notes were cleaned and expanded into comprehensive write-ups, which were uploaded into the qualitative data analysis software program ATLAS.ti version 7.1 (ATLAS.ti Scientific Software Development GmbH) along with the transcribed audio-recorded focus groups. Data were analyzed in two stages [13]. First-cycle coding allowed team members to summarize and reduce data from the notes and transcripts into broad, preliminary domains. Methods included deductive, structural coding with codes derived from the interview guide and inductive, descriptive coding using single word codes to describe the topic of a passage. Second-cycle coding allowed researchers to further reduce coded data into meaningful domains and themes. Team members established an interrater reliability rate of 80%.

## Results

### Veteran Participants

The majority of participants were older, white, non-Hispanic/non-Latino males, with a mean age of 63.5 years (SD 8.4), ranging from 43 to 83 years of age (data not shown). Most participants had at least a high school education with an annual income of US $25,001 or more (30/48, 63%); more than half (28/48, 58%) were married. Veterans were asked to list up to 10 of their chronic health conditions. Participants reported a mean of 6.5 (SD 1.89) conditions, ranging from 2 to 10 conditions (data not shown). Demographic data are presented in Table 1.

### Expert Panel

The expert panel included 34 representatives from 16 key VA operational offices and clinical disciplines including the VA’s Office of Mental Health; Office of Patient Centered Care and Cultural Transformation; Office of Rural Health; Office of Connected Health; Telehealth; My HealtheVet; VetLink Kiosks; Mobile Health; Human Factors; Pharmacy Informatics; Patient Education; and clinical disciplines such as primary care, specialty care, nursing, psychology, women’s health, and polytrauma.

### Overview of the VA Health Information Technology Systems Matrix

The VA HIT Systems Matrix is presented in Excel workbook spreadsheet format in Multimedia Appendix 1. There are four worksheets that present data: (1) access and function, (2) benefits and barriers, (3) system preferences, and (4) tasks. Within each worksheet, there is a two-axis inventory. The VA’s existing and emerging HIT platforms (eg, My HealtheVet, Mobile Health, VetLink Kiosks, Telehealth), My HealtheVet features (eg, Blue Button, secure messaging, appointment reminders, prescription refill, Veterans Health Library, spotlight, vitals tracker), and non-VA platforms (eg, phone/mobile phone, texting, non-VA mobile apps, non-VA mobile electronic devices, non-VA websites) are organized by row. Columns are titled with thematic and functional domains (eg, access, function, benefits, barriers, authentication, delegation, user tasks). Thematic and functional domains are presented in Textbox 1. Cells for each sheet include descriptions and details that reflect factors relevant to domains and the topic of each worksheet.

**Table 1:** Participant characteristics (N=48).

Characteristic		n (%)
**Gender**		
	Female	4 (8)
	Male	44 (92)
**Status**		
	Veteran	47 (98)
	Caregiver	1 (2)
**Education**		
	High school	7 (15)
	Some college/vocational	20 (42)
	Associate's degree	7 (15)
	College degree	7 (15)
	Graduate degree	7 (15)
**Race**		
	Caucasian/White	40 (83)
	African American/Black	5 (10)
	Native Hawaiian/other Pacific Islander	1 (2)
	American Indian/Alaskan Native	1 (2)
	Other American	1 (2)
**Ethnicity**		
	Hispanic or Latino	2 (4)
	Not Hispanic or Latino	45 (94)
	Declined to respond	1(2)
**Marital status**		
	Married	28 (58)
	Divorced	17 (35)
	Single/never married	3 (6)
**Annual income (US $)**		
	≤4999	3 (6)
	5000-10,000	1 (2)
	10,001-15,000	2 (4)
	15,001-25,000	7 (15)
	25,001-35,000	7 (15)
	35,001-45,001	6 (13)
	>45,001	17 (35)
	Declined to respond	5 (10)

**US Department of Veterans Affairs (VA) Health Information Technology Systems Matrix worksheet topics by column.**

**Access and function**

- Service availability
- Access
- Function

**Benefits and barriers**

- Benefits-expert panel members
- Barriers-expert panel members
- Benefits-veteran focus group participants
- Barriers-veteran focus group participants

**System preferences**

- Authentication
- Delegation
- Real-time synchronization
- Integration across platforms and with non-VA electronic resources
- Single sign-on
- Security
- Design

**Tasks**

- General tasks
- Communication with care team
- Laboratory test results
- Researching medical information
- Tracking health vitals
- Appointments
- Managing prescriptions

### Access and Function

This worksheet of the VA HIT Systems Matrix provides validated information from panel members about service availability, access requirements (eg, user group eligibility for different account types), and the function of each resource and feature. For example, eligibility and access requirements for the VA’s three My HealtheVet account types (basic, advanced, and premium) were not well understood by contributors and were often cited as barriers to use. Account types offer three different levels of access to patients’ health records, so understanding requirements can significantly impact a patient’s experience. These findings suggested that veterans required more comprehensive information about VA HIT function in order to appropriately utilize these tools to meet their self-care management needs.

#### Benefits and Barriers

This worksheet provides an overview of perceived VA HIT benefits and barriers from the panel and veterans’ perspectives. Benefits included 24-hour remote accessibility of appointment and prescription services, medical records, providers, and their ability to determine personal communication preferences. Panel members emphasized the efficiency and convenience of the resources and their benefit to patients. Barriers were related to accessibility, including limited access to requisite technologies or Internet connection and lack of available mobile technologies, lack of awareness of resources and how to use these resources, lack of accessibility to education, and navigation and system difficulties.

#### System Preferences

This worksheet addresses VA HIT system design preferences for authentication, delegation, synchronization, integration, sign-on, security, and interface design identified by panel and veterans’ groups. *Authentication* refers to the one-time identity authorization process required to obtain a premium-use My HealtheVet account for full system access. Many participants recommended a secure, online authentication process as opposed to the existing in-person requirement, which was perceived as inconvenient. The VA subsequently added an online option for authentication. Veterans also noted preference for mobile options, such as a My HealtheVet mobile app. *Delegation* refers to regulatory requirements, policy issues, and veteran preferences about surrogate account access (eg, who, timeframe, access level). Although unavailable at the time of the study, veterans felt that delegation represented a vital feature to the VA HIT system to better manage veterans’ care in partnership with informal caregivers and non-VA providers. *Real-time synchronization* and *integration across platforms and with non-VA electronic resources* details illustrate veterans’ preferences for an integrated, standardized, synchronized, and secure HIT system that integrates non-VA HIT across platforms such as kiosks and mobile apps. *Single sign-on* details addressed regulatory issues and veterans’ perceptions and concerns about the security and utility of federated credentialing, more commonly known as single sign-on. *Security* details provided by veteran participants indicate their clear concern for data privacy on VetLink Kiosks and mobile apps. Lastly, *design* details provided veteran input about the front-end user interface and experience when using VA HIT; most commonly, veteran participants stated a preference for simple “dashboard” designs that facilitated ease of use and continuity across resources and platforms.

#### Tasks

Veteran data clearly indicated that consumers used VA HIT to complete primary categories of tasks, including: (1) general tasks, (2) communication with care team, (3) laboratory test results/tests, (4) researching medical information, (5) tracking health vitals, (6) appointments, and (7) managing prescriptions. Data entries for these tasks provided veterans’ perspectives of the usefulness for utilizing each VA HIT resource and/or feature to complete the given task within different contexts. Due to the length and breadth of detail contained within the VA HIT Systems Matrix, the matrix document was structured to allow review and prioritization of content. The VA HIT Systems Matrix has search, filtering, and categorization options so content could be easily selected and compared (eg, to compare two or more resources).

### Veteran Recommendations for Educating Consumers About the VA Health Information Technology System

As previously stated, investments in education and marketing are necessary to promote veteran and provider access and sustained use of VA HIT. Thus, HIT Systems Matrix participants were asked to make consumer marketing and education recommendations for all VA HIT resources in order to address consumer confusion about resource access and function. Categories and recommendations are illustrated in Textbox 2.

**Veteran recommendations and strategies for educating consumers about US Department of Veterans Affairs (VA) health information technology (HIT).**

**Delivery**

- Involve veteran service organizations (eg, veterans of foreign wars) in educating veterans about VA HIT
- Target user groups who may require more assistance (eg, elders, traumatic brain injury)
- Provide peer-to-peer mentoring for sign-up instructions and updates
- Allow an educator to access veteran’s computer to help set up My HealtheVet, create icons, and teach veterans about My HealtheVet
- Have providers’ market services in person; provide brochures at the time of appointments with providers that explain key elements of VA resources

**Format**

- Provide multiple types of education to fit all learning styles (eg, paper-based, electronic, in person)
- Use graphics and pictures to augment text
- Provide a VA welcome package or mail a digital video transmitter (DVD/YouTube) when veterans request their ID cards.

**Communication**

- Send notifications about available technologies or changes to technologies (eg, secure messaging)
- Provide large print and display so veterans can read instructions easily in presentations (eg, PowerPoint)

**My Health***e* **Vet specific**

- Advertise tutorials/updates on VA sign-in page
- Integrate education on the My HealtheVet website
- Provide tutorials to learn more about My HealtheVet

**Troubleshooting**

- Establish a call desk to help veterans who are having trouble with aspects of VA HIT
- Inform veterans of the name and contact information for their My HealtheVet coordinator

## Discussion

Integrated HIT systems improve health care delivery and help veterans become active participants in their care and self-care management; HIT is essential to adequately address veterans’ health care needs [3]. Development and redesign in the VA must focus on the interactions and processes among patients, providers, administrators, organizational structures, and the technology itself to develop HIT resources that optimally meet consumer needs [3,14,15]. The data presented in the HIT Systems Matrix is relevant for VA administrators and developers as well as other large health care organizations seeking to document and organize their consumer-facing HIT resources.

The goal of this participatory study was to inform the VA’s vision of an integrated HIT system from the shared perspective of veterans, providers, and key stakeholders (eg, VA operational partners, clinicians). In alignment with the goals of the VA, the HIT Systems Matrix provides a descriptive blueprint for decision making and supports the ongoing development of a user-friendly HIT system that prioritizes increased access to personalized, proactive, and patient-driven virtual care. The utility of organizing and presenting information in the HIT Systems Matrix was threefold: (1) it allows users (eg, administrators and developers within and outside the VA) to view and interpret direct stakeholder and veteran input despite its organizing structure because it was designed to allow sorting and manipulation of data; (2) it provides a living, evolving document that can be shared at any stage of development and that can be updated as HIT systems evolve; and (3) it allows users of the tool to easily compare and contrast the characteristics of different HIT technologies and platforms as well as understand the VA HIT system as a single entity.

Although this study protocol and its HIT Systems Matrix product are useful in developing valuable knowledge to inform system improvements, this study has limitations. First, this study represented findings only relevant to the VA HIT systems and technologies; however, this study and its product may be useful for development and redesign of other tethered HIT systems in health care delivery. Second, current technological infrastructure capacity was not a primary focus and thus may have limited use of some findings although it should not limit the VA HIT vision of the future. Third, although our sample represented multiple stakeholder groups and was relatively small, it should be noted that participants were a representative, purposively sampled group and were comparable to sample sizes used in other qualitative mixed-methods studies [16]. As such, data presented in the matrix represent expert panel member and focus group participant reports only and in some instances may be incomplete. Fourth, we purposively recruited participants who were invested users of two or more platforms as we felt they could provide salient in-depth feedback. As such, we may have missed valuable data that may have represented noninvested users. Fifth, we purposefully included veterans with comorbid conditions because these individuals are more likely to leverage the use of electronic resources to manage their health care. As such, we may have missed valuable data that may represent healthier participants.

Future research should inform the ongoing development of VA’s vision for an integrated HIT system to include front-end patient user experiences and outcomes. Specifically, research should evaluate best practices for supporting consumers’ proactive and integrated use of VA HIT systems. Ongoing investigation in this area of research is also warranted to address identified barriers in the existing system and solutions to eliminating those barriers in the evolving VA HIT system. Issues for VA employees, including workload and workflow data, and organization-level research is needed to identify largescale infrastructural consequences and outcomes relevant to the supply and demand of the growing VA patient population. Finally, system preferences, such as single sign-on and delegation, merit further investigation to better understand the feasibility, acceptability, and usefulness of these features within the current and evolving VA HIT system across traditional (eg, personal computers) and emerging (eg, mobile technology) technologies. Delegation in particular has become increasingly important because the VA places more emphasis on engaging with community care providers and informal caregivers. The provision of comprehensive and consistent veteran health care rests on the veteran’s ability to securely and easily delegate access to medical records and virtual health services.

To our knowledge, this is one of the few published studies to aid in the development of an integrated system of HIT resources within a large health care system with nearly 4 million users (Veterans and Consumers Health Informatics Office, US Department of Veterans Affairs, 2016). Future research should inform the ongoing development of VA’s vision for an integrated HIT system to include front-end patient user experiences and outcomes. Specifically, research should evaluate best practices for supporting consumers’ proactive and integrated use of VA HIT systems.

Although this research lends itself to recommendations for future research, the authors’ aim in completing this work was to produce a useful resource to assist ongoing development, redesign, and research efforts. The VA is currently utilizing this tool to support multiple initiatives including the redesign of the patient portal, the design of an enterprise-wide delegation service, and has strategic communication plans to increase awareness and use of VA HIT. Beyond VA, other organizations can benefit from using a similar approach and may also find the matrix model useful as a template to enhance HIT analysis, strategy, planning, and use.

## Appendix

Multimedia Appendix 1 Department of Veterans Affairs Health Information Technology Systems Matrix.

## Abbreviations

HIT: health information technology
VA: Department of Veterans Affairs
